# Proliferation, Adult Neuronal Stem Cells and Cells Migration in Pallium during Constitutive Neurogenesis and after Traumatic Injury of Telencephalon of Juvenile Masu Salmon, *Oncorhynchus masou*

**DOI:** 10.3390/brainsci10040222

**Published:** 2020-04-08

**Authors:** Evgeniya V. Pushchina, Eva I. Zharikova, Anatoly A. Varaksin, Igor M. Prudnikov, Vladimir N. Tsyvkin

**Affiliations:** 1 Zhirmunsky National Scientific Center of Marine Biology, Far East Branch, Russian Academy of Sciences, 690041 Vladivostok, Russia; eva_1213@mail.ru (E.I.Z.); anvaraksin@mail.ru (A.A.V.); 2 Bogomoletz Institute of Physiology, National Academy of Sciences of Ukraine, 01024 Kyiv, Ukraine; specification77@yahoo.com (I.M.P.); malysh@biph.kiev.ua (V.N.T.)

**Keywords:** traumatic brain injury, adult neuronal stem cells, proliferation of cells, neuronal precursor cells, postmitotic neuroblasts, pallial part of telencephalon, masu salmon, GFAP, vimentin, doublecortin

## Abstract

A study of the lateral pallium in zebrafish and the visual tectum of the medaka revealed a population of adult neuroepithelial (NE) cells supported from the early stage of development to various postembryonic stages of ontogenesis. These data emphasize the importance of non-radial glial stem cells in the neurogenesis of adult animals, in particular fish. However, the distribution, cell cycle features, and molecular markers of NE cells and glial progenitors in fish are still poorly understood at the postembryonic stages of ontogenesis. Fetalization predominates in the ontogenetic development of salmon fish, which is associated with a delay in development and preservation of the features of the embryonic structure of the brain during the first year of life. In the present work, we studied the features of proliferation and the migration of neuronal precursors in the pallial proliferative zone of juvenile *Oncorhynchus masou*. The aim of the study is a comparative analysis of the distribution of glial-type aNSCs markers, such as vimentin and glial fibrillar acid protein GFAP, as well as the proliferation marker BrdU and migratory neuronal precursor doublecortin, in the pallial zone of the intact telencephalon in juvenile *O. masou* normal and after mechanical injury. The immunohistochemical IHC labeling with antibodies to vimentin, GFAP and doublecortin in the pallium of intact fish revealed single, small, round and oval immunopositive cells, that correspond to a persistent pool of neuronal and/or glial progenitors. After the injury, heterogeneous cell clusters, radial glia processes, single and small intensely labeled GFAP+ cells in the parenchyma of Dd and lateral part of pallium (Dl) appeared, corresponding to reactive neurogenic niches containing glial aNSCs. A multifold increase in the pool of Vim+ neuronal precursor cells (NPCs) resulting from the injury was observed. Vim+ cells of the neuroepithelial type in Dd and Dm and cells of the glial type were identified in Dl after the injury. Doublecortine (Dc) immunolabeling after the injury revealed the radial migration of neuroblasts into Dm from the neurogenic zone of the pallium. The appearance of intensely labeled Dc+ cells in the brain parenchyma might indicate the activation of resident aNSCs as a consequence of the traumatic process.

## 1. Introduction

The investigation of neurogenesis in adult animals is a significant area of neurobiology and developmental biology [1]. For the clarification of its mechanism, it is important to identify various types of cells with stem properties in the brain of adult animals, because of the heterogeneity of this process, which manifests a range of features, both at the level of individual constitutive neurogenic niches and in various species [1,2]. In the central nervous system (CNS) of amniotes, postembryonic populations of tissue-specific stem cells usually have a glial, radial glial, or astrocytic phenotype [3]. Neuroepithelial cells play a significant role in mammalian embryonic neurogenesis, while glial stem cells are the main source of neurons in the postembryonic stages of development [4]. In contrast to mammals, neuroepithelial-like stem cells/progenitor cells are present in the brain of teleosts throughout life [4,5].

In mammals, the distribution of adult neuronal stem cells (aNSCs) is mainly limited to the forebrain [1]. They originate from embryonic radial glial cells [6] and do not directly produce neurons. Instead, they first produce intermediate progenitor cells [7], which are highly heterogeneous in terms of active cyclicity or dormancy [8] and are expressed molecular markers [9,10]. In aNSCs studies on zebrafish, radial glial NSCs and intermediate progenitors have been identified [3,5]. However, the results of studies on a medaka [4] did not confirm the presence of such cells in the tectum of this fish species. Neuroepithelial cells (NE) were identified in the medial pallium during development and such cells can arise directly from embryonic precursors [11] in the areas of proliferation of the dorsal telencephalon.

The brain of an adult fish is a site of continuous intense proliferative activity, not limited only to the telencephalon [12]. In some areas of proliferation in the brain, tissue-specific stem cells have a neuroepithelial phenotype rather than radial glial characteristics [5,13]. Studies on juvenile salmon fish showed that in the cerebellum of *O. masou* [13] and in the mesencephalic tegmentum of the chum salmon *Oncorhynchus keta* [14], most aNSCs have a neuroepithelial phenotype. Such cells divide symmetrically, constantly increasing the pool of neural precursors, which subsequently form neurons, glia-like cells, and ependymal cells that form the CNS [7]. Of particular importance in the postembryonic CNS development of salmon fish is the fetalization process, which is associated with a delay in the development and retention of the features of the embryonic organization of the brain [15]. Studies on medaka brain have also shown that in optic tectum, as a part of proliferative populations, aNSCs are actually neuroepithelial-like stem/progenitor cells and are not radial glial cells [4]. Similar results were obtained in other studies on fish brain, in particular, optic tectum [16], mesencephalic tegmentum [14], and cerebellum [13,17].

A study of the lateral pallium in *D. rerio* revealed a population of adult NE cells [18], supported from an early stage of development to various postembryonic stages of ontogenesis. All these data emphasize the importance of non-radial glial stem cells in the neurogenesis of adult animals such as fish. However, the distribution, cell cycle features, and molecular markers of NE cells and glial precursors in fish are still poorly understood at the postembryonic stages of ontogenesis. 

Salmon fish are an ancient group of ray-finned fish, with a large number of undifferentiated elements in the brain and high proliferative potential. Their brains retain the signs of ancestral organization throughout their life and are characterized by a developmental delay with long-term preservation of the features of the embryonic structure. This makes juvenile salmon fish a convenient model for studying the processes of postembryonic development of the central nervous system, investigation of properties of adult stem cells/progenitor cells, as well as cell migration processes during constitutive neurogenesis and after traumatic injury. Currently, the development of the adult brain in the Pacific salmon population, unlike in the Atlantic salmon, has not been practically studied.

In the present work, we studied the features of proliferation and migration of neuronal precursors in the pallial proliferative zone of juvenile *O. masou*. It has been shown that any single immunolabeling method has limitations for the study of neurogenesis, because the phenotype of various neuro- and gliospecific precursors is usually more complex and contains more than one IHC marker. Nevertheless, given that the brains of Pacific salmon are still poorly studied and little is known about the populations of proliferating pallial cells, as well as the distribution of tissue-specific markers of neuronal and glial differentiation, we performed a comparative analysis of the distribution of glial-type aNSCs markers (vimentin and GFAP), the proliferation marker (BrdU), and migratory neuronal precursors (doublecortin) in the pallial zone of the intact telencephalon in juvenile *O. masou* and after a mechanical injury.

## 2. Material and Methods

### 2.1. Experimental Animals

We used 40 juvenile (one-year-old) specimens of *Oncorhynchus masou*, with a body length of 11–13.5 cm and a weight of 35–50 g. The animals were obtained from the Ryazanovka experimental fish hatchery in 2019. Fish were kept in an aquarium with aerated fresh water at a temperature of 16–17 °C and fed once a day. The light/dark cycle was 14/10 h. The content of dissolved oxygen in water was 7–10 mg/dm^3^, which corresponds to normal saturation. All experimental manipulations with animals were carried out in accordance with the rules regulated by the Commission on Biomedical Ethics, Zhirmunsky National Scientific Center of Marine Biology (NSCMB), Far East Branch, Russian Academy of Sciences (FAB RAS) (approval # 1-260220 from Meeting No. 1 of the Commission on the biomedical ethics of NSCMB FEB RAS, 26 February 2020). Fish were anesthetized in a 0.1% solution of tricaine methanesulfonate MS222 (Sigma, St. Louis, MO, USA) for 10–15 min.

After the anesthesia, the intracranial cavity of the immobilized animal was perfused with a 4% paraformaldehyde solution in 0.1 M phosphate buffer (pH 7.2), using a syringe. After prefixation, the brain was removed from the cranial cavity and fixed in a 4% paraformaldehyde solution for 2 h at 4 °C. Then, it was kept in a 30% sucrose solution at 4 °C for 2 days (with a five-fold change of solution). Thick (50 μm) frontal sections of the *O. masou* brain were made on a Cryo-star HM 560 MV freezing microtome (Carl Zeiss, Oberkochen, Germany), mounted on polylysine slides (Biovitrum, Russia), and dried.

### 2.2. Experimental Damage to the Brain by the Kishimoto Method 

Animals were anesthetized in a cuvette with a 0.1% solution of tricaine methanesurfanate (MS-222, Aldrich, Sigma, St. Louis, MO, USA), for 10 min at room temperature. Using a sterile needle (BD Bioscience, Cat. No 305109), a mechanical damage was applied to the dorsolateral quadrant of the right hemisphere of the telencephalon to a depth of 1 mm [19]. Immediately after the damage was applied, the animals were released into the aquarium for recovery and further monitoring.

After a damaging effect in the region of the telencephalon, video monitoring of changes in locomotor and behavioral activities in fish in the experimental group was performed for 1 h. No significant changes were found in locomotor activity in animals with the brain injury, compared with the control group. A small hematoma of 1–2 mm in size was clearly visualized in the area of injury.

### 2.3. Preparation of Material for Immunohistochemical Studies

After 1 week, the animals were removed from the experiment and were euthanized by rapid decapitation. The brain was perfused with a 4% parapharmaldehyde solution in 0.1 M phosphate buffer (pH 7.2). After prefixation, the brain was removed from the cranial cavity and fixed in the same solution for 2 h at 4 °C. Then, it was washed for two days in a 30% sucrose solution at 4 °C. Serial frontal 50-μm-thick sections of the *O. masou* brain were made on a freezing microtome (Cryo-star HM 560 MV, Oberkochen, Germany).

### 2.4. Experimental BrdU Labeling

To study the proliferation processes in intact animals and 1 week after a traumatic damage to the telencephalon of juvenile *O. masou*, IHC labeling of BrdU was used. The fish were subjected to general anesthesia by immersion in a tank with 2% MS 222 (St. Louis, LO, USA). A 1-mm-deep traumatic injury was inflicted in the parasagittal direction of the telencephalon. In the control group of animals and after a telencephalon injury (*n* = 5 for each group), an intraperitoneal injection of 10 mg/mL BrdU solution (Sigma-Aldrich, St. Louis, LO, USA) at a dose of 20 μL/g body weight was injected to animals, simultaneously with brain damage. After a 7-day period, the fish brain was removed for further preparation. Animals of the control group (*n* = 5) received only BrdU injection. Immediately after the traumatic injury, the animals were released into a common tank with fresh water, for their recovery and further monitoring.

For further IHC studies, fish were anesthetized in a cuvette with a 0.1% solution of tricaine methane sulfonate MS-222 for 5 min, the skull was opened, and the brain was removed. Then, the whole brain was embedded into paraffin according to the generally accepted technique [20], after which serial transverse 7-μm-thick sections of the brain were made and mounted on polylysine slides. Then, the sections were dehydrated according to the standard histological protocol [13]. At the last stage, they were washed in distilled water for 3 min. Sections were further processed according to the protocol for IHC labeling of BrdU [21]. To untwist the double-stranded structure of DNA, acid hydrolysis was performed. Brain sections were incubated in 1 M HCl for 10 min on ice, and then incubated in 1 M HCl for 10 min at room temperature, then for 20 min at 37 °C. Immediately after incubation with acids, the sections were neutralized in a 0.1 M borate buffer for 10 min at room temperature and washed three times in the phosphate buffer PBS (pH 7.4), 0.1% Triton X-100, three times, with 5 min per wash. A 1% hydrogen peroxide solution on 0.1 M phosphate buffer was applied to the sections (pH 7.2). The sections were then incubated for 20 min at room temperature and washed thrice in 0.1 M phosphate buffer for 5 min per wash. Subsequently, the sections were incubated with the anti-bromodeoxyuridine/BrdU monoclonal mouse antibody (1:200; clone SPM166; Novus Biologicals, Littleton, MA, USA), at room temperature for 30 min and then washed in three shifts of 0.1 M phosphate buffer for 5 min per shift. To visualize IHC labeling, a standard Vectastain Elite ABC kit (Vector Laboratories, Burlingame, CA, USA) was used according to the manufacturer’s instructions. The red substrate (VIP Substrate Kit, Vector Labs, Burlingame, CA, USA) was used for the visualization of the IHC reaction. The sections were dehydrated according to a standard procedure and enclosed under coverslips in the Bio-optica medium (Milano, Italy) for histological sections.

### 2.5. Immunohistochemical Detection of Doublecortin, Vimentin and Glial Fibrillar Acidic Protein

Molecular markers of astrocytic glia were used in the study: glial fibrillar acidic protein (GFAP) and vimentin [22]. The doublecortin protein associated with microtubules and expressed by immature neurons was used as a marker of neuronal migration [23]. To study the expression of doublecortin, vimentin, and GFAP in the telencephalon of juvenile *O. masou*, immunoperoxidase labeling was used on frozen, free-floating sections of the brain. Monoclonal mouse antibodies to GFAP (GF5 Catalog No. ab10062), vimentin, and doublecortin from Abcam (Cambridge CB2 0AX, UK) at a dilution of 1: 300 were used on 50-μm-thick transverse sections incubated in situ at 4 °C for 48 h. The assessment of protein expression was carried out 7 days after the traumatic injury to telencephalon. 

For visualization of immunohistochemical (IHC) labeling, the standard ABC complex Vectastain Elite ABC kit (Vector Laboratories, Burlingame, CA, USA) was used in accordance with the manufacturer’s recommendations. A red substrate was used to identify reaction products (VIP Substrate Kit, Vector Labs, Burlingame, CA, USA). The brain sections were mounted on slides with a polylysine coating (BioVitrum, St. Petersburg, Russia) and left to dry completely. To identify immuno-negative cells, the brain sections were further stained with 0.1% methyl green solution (Bioenno, Lifescience, CA, USA, Cat # 003027). The color development was monitored under a microscope, and washed with distilled water for 10 s, after which they were differentiated for 1–2 min in a solution of 70% alcohol and then 10 s in 96% ethanol. The brain sections were dehydrated according to the standard method: two changes of xylene were used, 15 min each, and then they were embedded in the Bio-optica medium (Milano, Italy) under coverslips.

To assess the specificity of the immunohistochemical reaction, the negative control method was used. Instead of primary antibodies, brain sections were incubated with a 1% solution of non-immune horse serum for 1 day and treated as sections with primary antibodies. In all control experiments, the immunopositive reaction was absent.

### 2.6. Microscopy

For visualization and morphological analysis, we used a motorized inverted microscope of a research class with a fluorescent module and an enhanced contrast attachment when working with Axiovert 200 M luminescence with an ApoTome module (Carl Zeiss, Oberkochen, Germany). Microphotographs of the preparations and material analysis were performed using the Axio Vision program (Carl Zeiss, Oberkochen, Germany). The measurements were carried out with magnification 100×, 200×, 400× and 630× in several randomly selected fields of view for each area of study. The number of immunolabeled cells in the field of view was counted at a magnification of 200×. Morphometric analysis of the parameters of cell bodies (measurements of the greater and lesser diameters of the neurons’ soma) was performed using the Axiovert 200 M microscope software (Carl Zeiss, Oberkochen, Germany). Micrographs of the brain sections were taken with an Axiovert 200 digital camera. The material was processed using the Axioimager program (Carl Zeiss, Oberkochen, Germany).

### 2.7. Statistical Analysis

Quantitative processing of the material was performed using the Descriptive Statistics programs available in the Microsoft Excel 2010, Statistica 12 packages, and STATA statistical software (StataCorp. 2012. Stata Statistical Software: Release 12. College Station, TX: StataCorp LP, USA). Prior to the experiments, we performed a statistical analysis based on the variations in the measured parameters in our previous research [15] and determined that we needed a group of at least 4 animals to achieve the statistical confidence at 95%. To make sure that we reach a group size of 4 and, at the same time, reduce the use of animals to a minimum, we aimed at total of 5 animals per experimental group. The distribution density and dimensional characteristics of the cells were estimated using the methods of variation statistics. To quantify the results, mean values and standard deviation (M ± SD) were found and analyzed with the SPSS software (version 16.0; SPSS Inc., Chicago, IL, USA). All variables measured in groups were compared using Student–Newman–Cales test or a one-way analysis of variance (ANOVA, Chicago, IL, USA) with Bonferroni correction. Values at *p* ≤ 0.05 were considered statistically significant.

## 3. Results

### 3.1. Experimental Labeling of Brdu in the Intact Pallium of Juvenile O. masou and after a Traumatic Injury

**Figure 1:**
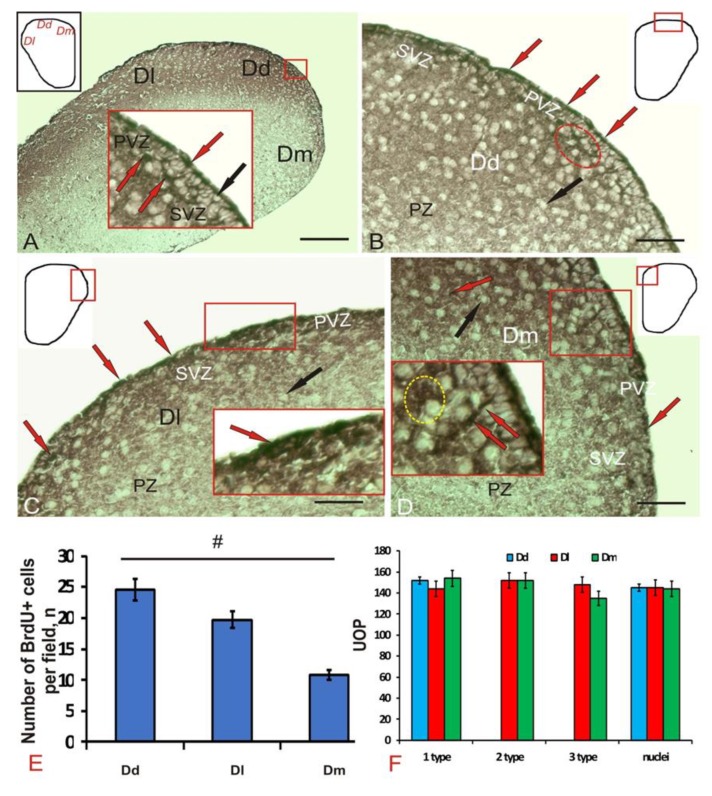
Representative image of BrdU-immunolabeling in the pallial region of the telencephalon of an intact juvenile *Oncorhynchus masou*. (**A**) General view of the telencephalon, the pictogram shows the zones of the dorsal telencephalon (pallium), Dd-dorsal, Dm-medial, Dl-lateral, the inset (outlined by a red rectangle) shows a fragment including PVZ-periventricular zone and SVZ-subventricular zone, BrdU+ cells are shown by red arrows, BrdU-cells are indicated by black arrows; (**B**) dorsal zone (Dd) at a higher magnification, accumulation of BrdU+ nuclei in the red oval, PZ-parenchymal zone of the telencephalon, for other designations, see Figure 1A; (**C**)-lateral zone (Dl) at a higher magnification, migrating tangentially elongated BrdU+ cells are shown by red arrows, BrdU+ cells in the surface layer of the PVZ are shown in the inset (in the red box); (**D**) medial zone (Dm), the group of radially migrating BrdU+ cells is shown in the inset (in the red rectangle), the cluster of BrdU+ nuclei in the yellow oval. Immunohistochemical labeling of 5-brom-2-deoxy-uredine. Scale bar: in (**A**) 200 μm, (**B**–**D**) 100 μm. (**E**) The comparative distribution of BrdU+ cells in various regions of intact pallium. (*n* = 5 in each group; #-significant intergroup differences). One-way analysis of variance (ANOVA); (**F**) the optical density of BrdU immunolabeling in cells of various types and nuclei (M ± SD) of Dd, Dl, and Dm of pallium of juvenile *O. masou*, UOP—the units of optical density.

At 1-week post-injury, after experimental incorporation of BrdU into the *O. masou*, the number of labeled nuclei and cells was estimated in telencephalon. In the telencephalon of *O. masou*, we examined the dorsal (Dd), lateral (Dl), and medial (Dm) pallial regions (Figure 1A). Immunoperoxidase labeling in the periventricular zone revealed that densely stained BrdU+ nuclei and cells (Figure 1A, inset, Table 1), immunonegative cells of the subventricular (SVZ) and parenchymal (PZ) zones, as a rule, were not stained. The densitometric characteristics of BrdU-immunolabeling in cells and nuclei of pallial brain regions of juvenile *O. masou* are given in Table 1. In control animals, BrdU+ nuclei had similar morphological characteristics, and the cells had a rounded or oval shape and a high BrdU-labeling intensity (Table 1).

In the dorsal zone (Dd), we found the largest number of small BrdU+ cells located in the surface and deeper parts of the periventricular zone (PVZ) (Figure 1B). The sizes of immunopositive cells and nuclei are shown in Table 1. BrdU-labeled small cells in Dd were of the same rounded or oval shape (Figure 1B, Table 1). Small clusters of nuclei were found in the SVZ (Figure 1B, Table 1). In Dl, the number of BrdU+ cells was lower than in Dd; however, the population of immunopositive cells was heterogeneous (Figure 1C, Table 1). Three types of cells were revealed in Dl: small, oval, and superficially located elongated cells oriented tangentially not found in other areas (Figure 1C, inset). The smallest number of BrdU+ cells and nuclei was recorded from Dm; however, elongated immunopositive cells were found in deeper subventricular layers in this region (Figure 1D, inset). Among BrdU+ cells in Dm, as in Dl, 3 types were distinguished (Table 1). 

Cells were detected in all areas of the pallial zone of the juvenile *O. masou* telencephalon, the number of which was maximum in Dd and minimum Dm (Figure 1E). Significant intergroup differences (*p* < 0.05) of the average number of cells per view field were observed between these two regions of the *O. masou* pallium (Figure 1E). In Dl, the number of BrdU+ nuclei and cells was lower than in Dd and higher than in Dm (Figure 1E); however, no significant intergroup differences with other groups were revealed. The densitometric parameters of BrdU+ nuclei and cells of various types in the pallial region of the *O. masou* indicate their intensive labeling (Figure 1F, Table 1).

**Figure 2:**
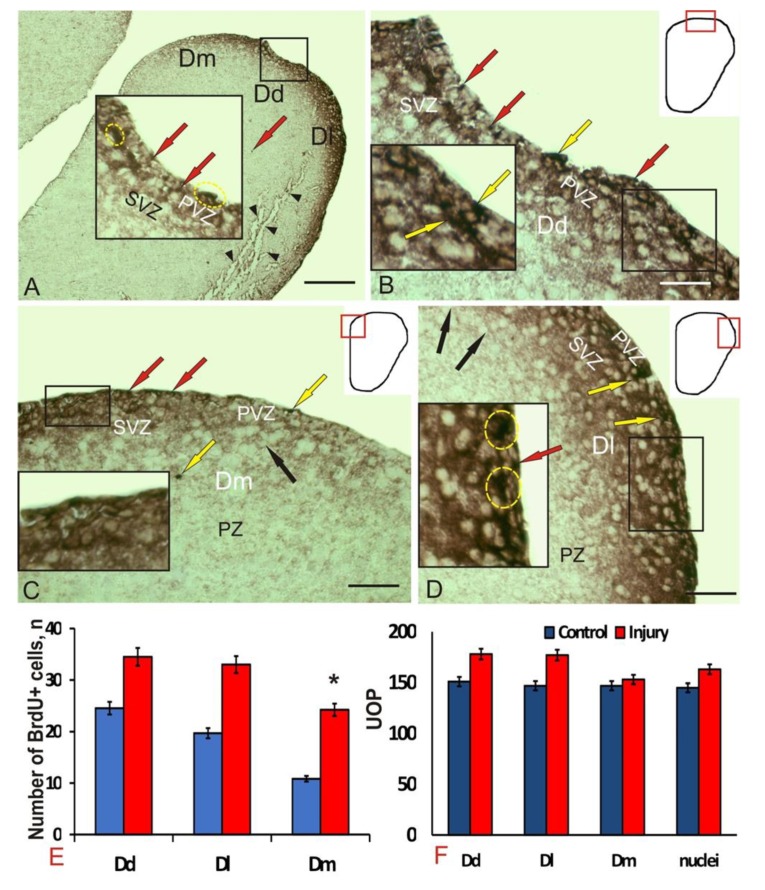
Representative image of BrdU-immunolabeling in the pallial region of the telencephalon of the juvenile *Oncorhynchus masou* 1 week after the traumatic injury of the telencephalon. (**A**) On the left is a general view of the damaged hemisphere of the telencephalon; on the right, a fragment is partially shown of the intact hemisphere of the telencephalon; Dd—the dorsal zone; Dm—the medial; Dl—lateral; shown by red arrows, clusters of BrdU+ cells in PVZ (circled by a yellow dotted line), BrdU+ cells and nuclei in the area of injury are shown by black triangular arrows; (**B**) dorsal zone (Dd) at a higher magnification, the accumulation of BrdU+ cells in the PVZ is shown by yellow arrows (inset); (**C**) the medial zone (Dm) at a higher magnification, BrdU+ nuclei are shown by red arrows, yellow arrows show large BrdU+ cells in PVZ and PZ, the inset shows a PVZ fragment with a multilayer distribution of BrdU+ cells; (**D**) lateral zone (Dl) at a higher magnification, containing numerous clusters of BrdU+ cells in the PVZ, are shown by yellow arrows, in the inset an enlarged fragment of BrdU—immunopositive conglomerates (circled by yellow ovals), BrdU—cells are shown by black arrows; Immunohistochemical labeling of 5-brom-2-deoxy-uredine. Scale bar: in (**A**) 200 μm, (**B**–**D**) 100 μm. (**E**) The quantitative ratio of BrdU+ cells in intact animals (control group) and 1 week after traumatic injury (*n* = 5 in each group, * *p* ≤ 0.05—significant difference from control groups. Student–Newman–Cales test; (**F**) the ratio of the optical density of BrdU-immunolabeling in the cells and nuclei of intact animals (control) and 1 week after traumatic injury (M ± SD) in Dd, Dl and Dm of the pallium of juvenile *O. masou*, UOP—the unit of optical density.

Seven days after the traumatic injury, the number of BrdU+ cells and nuclei in all pallial zones increased (Figure 2A). In the PVZ of Dd, Dl, and Dm, the number of morphologically heterogeneous immunopositive nuclei (Figure 2A, inset) of two-dimensional types significantly increased (Table 1). In Dd, 2 types of labeled cells were detected (Figure 2B), in contrast to control animals (Table 1). In Dm, an increase in small intensely labeled cells in PVZ was recorded (Figure 2C); in comparison to control; there was a decrease in the total number of morphological cell types in Dm (Table 1). Labeled cells and nuclei formed small clusters (Figure 2C, inset); the tangential migration patterns detected in control animals were not found after the injury. In Dl, the number of BrdU+ cells decreased to two types compared to the control (Figure 2D, Table 1). Single oval cells were found in PVZ and parenchymal layers (PZ); smaller BrdU+ cells formed small clusters (Figure 2D, inset). Marked nuclei of two types (Table 1) were found in the surface layers of the PVZ and SVZ (Figure 2D). The number of BrdU+ labeled cells and nuclei in Dm significantly increased (*p* < 0.05) compared with the control (Figure 2E). The intensity of BrdU-immunolabeling after injury also increased by 10%–15% in the cells and nuclei of all areas of the pallial zone (Figure 2F).

Thus, after the traumatic injury to the telencephalon, an increase in the immunolabeling intensity and the number of BrdU+ cells and nuclei was observed in all areas of the juvenile *O. masou* pallium. Significant differences from the control group were identified in Dm. After the traumatic injury, the morphological composition of the cells involved in proliferation changed. An additional type of BrdU+ cells appeared in Dd, while in Dl and Dm, on the contrary, there was a decrease in the number of labeled cell types to two and one, respectively (Table 1). In all the pallial areas, smaller BrdU+ nuclei appeared after the injury, in contrast to the control.

### 3.2. Immunohistochemical Gfap Labeling in the Intact Juvenile O. masou Pallium and after Traumatic Injury

**Figure 3:**
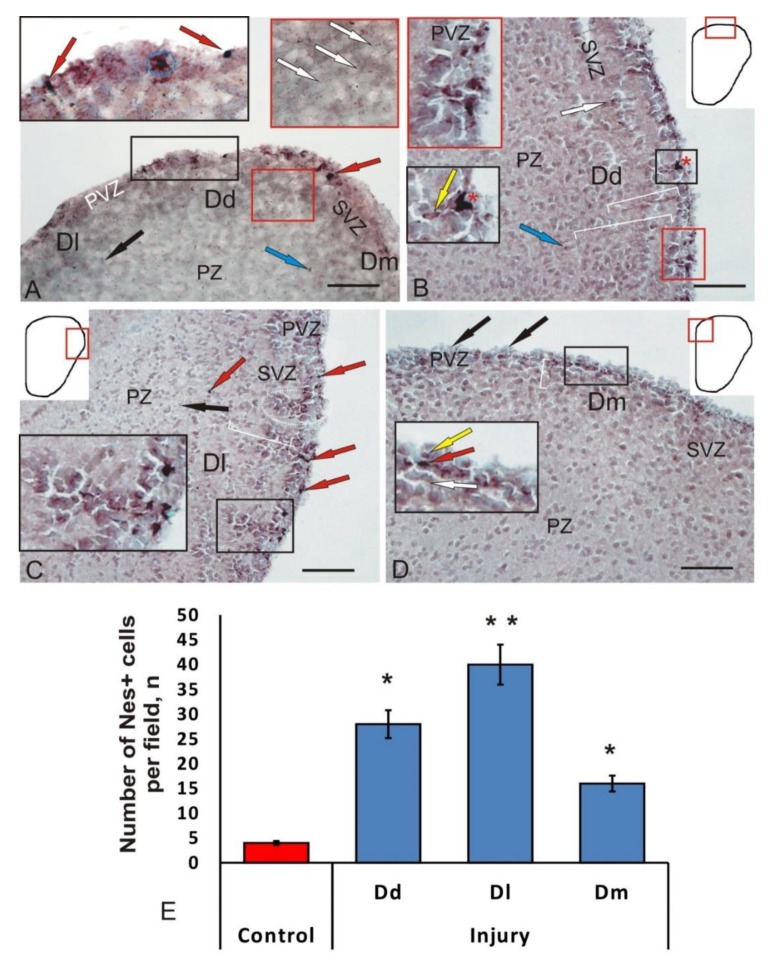
Representative image of GFAP-immunolabeling in the pallial region of the intact telencephalon (**A**) of juvenile *Oncorhynchus masou* and 1 week after traumatic injury (**B**–**D**) of the telencephalon. (**A**) Localization of GFAP in intact pallium, immunopositive cells (red arrows) and their clusters (blue dashed line) in PVZ (inset in the black rectangle) and radial glia fibers (white arrows) in the SVZ (inset in the red rectangle); (**B**) GFAP+ cells complexes (inset in red rectangle) and clusters (inset in black rectangle) of intensely GFAP-labeled cells (red star) and moderately GFAP-labeled cell (yellow arrow) in PVZ of Dd, 1 week after damage to the telencephalon, radial glia fibers bounded by white lines, GFAP+ granules (blue arrow); (**C**) post-traumatic reorganization of GFAP immunopositivity in Dl, GFAP+ heterogeneous complexes (in a white dashed oval) in PVZ and SVZ (an enlarged fragment is shown in the inset), alternating with immuno-negative regions, radial glia fibers are bounded by a white segment, GFAP+ cells are shown by red arrows; (**D**) heterogeneous GFAP+ cells after trauma in the PVZ of Dm (inset), moderately labeled (yellow arrows), intensely labeled (red arrows) and radial glia (white arrows); (**E**) quantitative ratio of GFAP+ cells in intact animals (control group) and 1 week after traumatic injury to the telencephalon (*n* = 5 in each group, * *p* ≤ 0.05; ** *p* ≤ 0.01; significant difference from control groups). Student–Newman–Cales test. Immunohistochemical labeling of glial fibrillar acidic protein in combination with methyl green staining. Scale bar: in (**A**) 200 μm, (**B**–**D**) 100 μm.

Several types of GFAP-immunopositive cells that differed in morphological characteristics and the intensity of immunolabeling in all areas of the pallium were identified in intact animals (Table 2). Among the intensively labeled cells (of the first type), several size groups were distinguished (Table 2), which obviously represented various stages of growth of GFAP+ glial progenitors. Among these cells, single intensely labeled cells were found (Figure 3A, black box). The other group included moderately GFAP-labeled cells, morphologically heterogeneous (Table 2), with a centrally located negative nucleus, surrounded by a rim of labeled cytoplasm (Figure 3A, black box). Such cells usually formed small conglomerates of 3–5 densely arranged elements. In some cases, it was possible to find weakly labeled radial fibers and small weakly or moderately labeled granules, located in the preventricular and subventricular layers (Figure 3A, red box). No GFAP+ cells and fibers were found in the deep parenchymal layers, but there were single, weakly labeled positive granules (Figure 3A).

After the injury in the Dd of the telencephalon, *O. masou* intensely GFAP-labeled, elongated or rounded cells revealed (Figure 3B, inset). The most intense labeling was observed in elongate conglomerates of cells located in the SVZ, under the layer of negative neuroepithelial cells (Figure 3B, Table 2). In the SVZ, immediately below those that were intensely labeled, there were single moderately labeled cells with a centrally located negative nucleus (Figure 3B, Table 2). Accumulations of GFAP+ cells were often limited to bundles or single radial fibers up to 195 μm in length (Figure 3B).

The parameters of GFAP+ cells in the control animals and in the post-traumatic period in Dd were different (Table 2); as a result of the injury, larger, intensely and moderately labeled cells appeared. Cells containing subcellular immunopositive inclusions were identified in PVZ (Figure 3B, inset). Separate GFAP+ small, intensely labeled cells and granules were found in the parenchymal layers (Figure 3B). As a result of the injury, the number of GFAP+ cells in the view field in Dd increased by 4 times (Figure 3E).

GFAP+ cell complexes, including elements located in the territory of the PVZ and SVZ, appeared in Dl after the mechanical injury (Figure 3C, Table 2). In such complexes, the following elements were distinguished: components located in the PVZ, represented by intensely and moderately labeled cells; a migratory population of SVZ cells (Figure 3C, inset). The entire complex was permeated with GFAP+ radial glia fibers (Figure 3C). GFAP+ complexes alternated with immuno-negative regions that did not contain immunolabeled elements. The morphological heterogeneity of GFAP+ cells in PVZ and PZ increased, and smaller cell forms appeared (Table 2). In the deep parenchymal layers of Dl, single intensively labeled GFAP small cells were observed (Table 2). The length of the GFAP+ complexes in the radial direction was ca. 100 μm; 3–4 of them were observed in each field of view. Individual intensely labeled radial glia cells were recorded from the PVZ (Figure 3C). The labeling intensity of such cells varied from moderate to high (Table 2).

After the injury, similar structural changes were detected in Dm, but they were less pronounced than in Dd and Dl (Figure 3D). In PVZ, a few intensely and moderately labeled large cells were detected (Table 2). Compared to the control group (* *p* < 0.1), a threefold increase in the number of GFAP+ cells was observed in Dm (Figure 3E).

Thus, as a result of the injury, structural changes were found in the periventricular and subventricular regions in Dl, which resulted in the appearance of reactive structural complexes, including a heterogeneous population of GFAP+ cells and radial glia. The pronounced topological nature of such complexes, the differentiated organization of various types of cells in them, and the high hierarchy in the ratio of individual elements suggest that these complexes correspond to reactive neurogenic niches containing glial progenitors specific for postembryonic neurogenesis, reactivating as a result of the traumatic process.

### 3.3. Immunohistochemical Labeling of Vimentin in the Intact Juvenile O. masou Pallium and after Traumatic Injury

**Figure 4:**
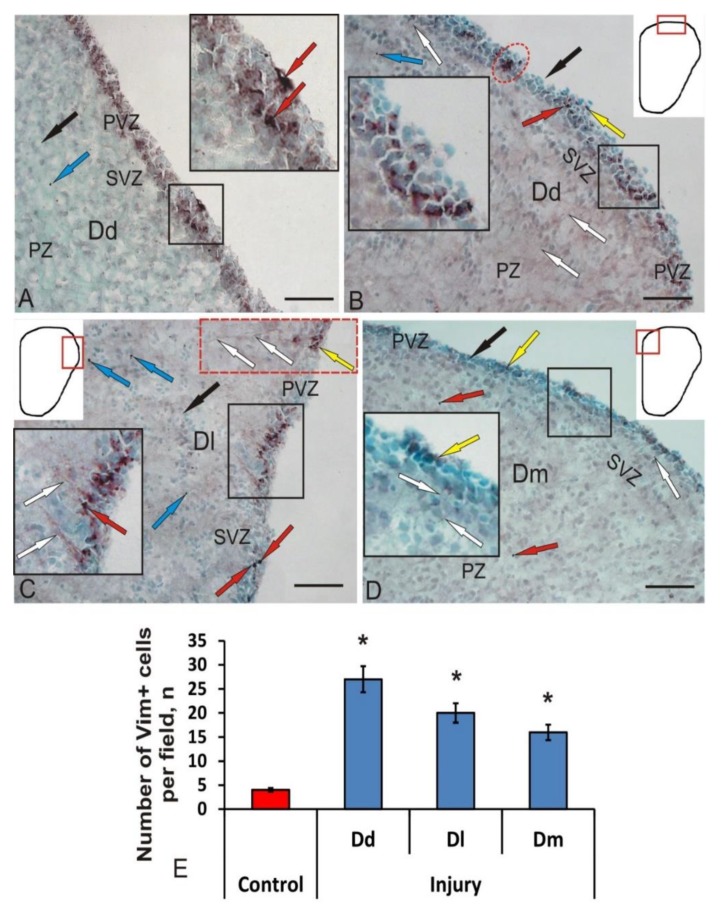
Representative image of Vim-immunolabeling in the pallial region of the intact telencephalon (**A**) of juvenile *Oncorhynchus masou* and 1 week after traumatic injury (**B**–**D**) of the telencephalon. (**A**) Localization of Vim in intact pallium, immunopositive cells (inset, red arrows) and granules (blue arrow) in the parenchymal zone (PZ), Vim- cells (black arrow); (**B**) Vim+ cell complexes (inset) and clusters (red dashed oval) of intensely Vim-labeled cells (red arrow) and moderately Vim-labeled cell (yellow arrow) in PVZ of Dd, 1 week after damage to the telencephalon, radial glia fiber (white arrows), Vim+ granules (blue arrow); (**C**) post-traumatic reorganization of Vim-immunopositivity in Dl, Vim+ heterogeneous complexes in PVZ and SVZ (an enlarged fragment is shown in the inset), alternating with immuno-negative regions, radial glia fibers are limited by a red dashed rectangle, intensely Vim-labeled cells are shown by red arrows, moderately Vim- labeled cells (yellow arrow); (**D**) weakly labeled Vim+ cells after injury in the PVZ of Dm (inset), moderately labeled cells (yellow arrow), intensely labeled granules (red arrows), Vim- cell (black arrow) and radial glia (white arrow); (**E**) the quantitative ratio of Vim+ cells in intact animals (control group) and 1 week after traumatic injury to the telencephalon (*n* = 5 in each group, * *p* ≤ 0.05; significant difference from control groups). Student–Newman–Cales test. Immunohistochemical labeling of vimentin in combination with methyl green staining. Scale bar: in (**A**) 200 μm, (**B**–**D**) 100 μm.

Products of normal IHC reaction after vimentin labeling were detected in the cells of the periventricular region, in the form of small granules located in different parts of the bodies of immunopositive cells (Figure 4A). The pattern of cell immunolabeling varied from fine to coarse. The intensity of vimentin immunolabeling in PVZ cells was weak or moderate (Figure 4A, Table 2). Single intensely labeled cells, as a rule, formed small dense clusters (Figure 4A, inset), or were located in the form of a multilayer stratum in the PVZ (Figure 4A). Along with labeled intracellular granules, separate intensely labeled elongated cells were identified in the PVZ (Figure 4A, inset, Table 2). In the parenchymal zone of the brain, we found a few Vim+ granules, located both in the intercellular space and inside cells (Figure 4A).

After the traumatic injury to the pallial region, we detected an increase in vimentin expression in some cells and small clusters of PVZ. In intensively labeled cells of Dd, as a rule, the nucleus was not visualized (Figure 4B, Table 2). Separately located Vim+ cells often had long, radially oriented thin processes (Figure 4B, Table 2). In moderately labeled cells, we identified Vim+ granules concentrated at one of the cell poles (Figure 4B, Table 2). Longer Vim+ cytoplasmic inclusions were also found sometimes (Figure 4B, inset). Superficially located neuroepithelial cells were Vim- (Figure 4B). In the brain parenchyma, single, small Vim+ cells (Table 2), as well as a few diffusely arranged Vim+ granules, were observed (Figure 4B).

In Dl, we identified clusters of Vim+ cells that extended to a distance of 130 μm (Figure 4C, inset). Vim+ small granules, Vim+ radial glia cells, and Vim- cells (Figure 4C, inset) were clearly distinguished in the composition of clusters. In the SVZ, it was possible to find focuses of convergence of Vim+ RG fibers and multidirectional processes organized in the form of bundles. Immunopositive aggregations were separated by small areas containing negative cells (Figure 4C).

In other cases, we observed small, intensely Vim-labeled cells containing processes (Table 2). We attributed this type of cell to the postembryonic population of tissue-specific aNSC stem cells with a radial glial phenotype (Figure 4C, inset). Along with intensely Vim-labeled cells, we observed moderately Vim labeled cell clusters in PVZ (Figure 4C, inset, Table 2). Such clusters contained Vim+ RG, whose fibers extended from SVZ to deeper layers of PZ (Figure 4C, inset). Along the RG fibers and in the region of their endings, individual highly Vim-immunopositive granules could be found (Figure 4C). In PZ, individual small intensely Vim-labeled cells and numerous Vim-immunopositive granules with intracellular localization were identified (Figure 4C; Table 2).

After the injury, individual moderately or weakly Vim+ few cells located in the PVZ were detected in Dm (Table 2). Cells had immunopositive inclusions scattered in the cytoplasm (Figure 4D). Small Vim-labeled RG fibers extending in PZ were also identified (Figure 4D, inset). Vim+ cells had a rounded or oval shape (Table 2). The optical density of immunopositive inclusions was weak (Table 2). In Dm, the number of Vim+ cells (Figure 4E) and the intensity of immunopositive inclusions after the injury were minimal in comparison with those in Dd and Dl (Figure 4D).

### 3.4. Immunohistochemical Labeling of Doublecortin in the Intact Juvenile O. masou Pallium and after Traumatic Injury

**Figure 5:**
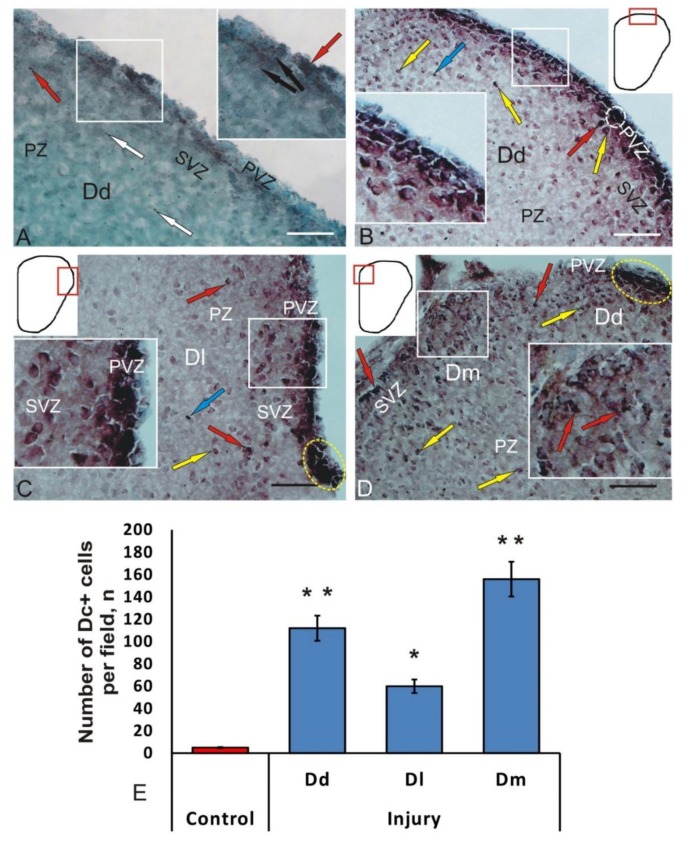
Representative image of doublecortin DC-immunolabeling in the pallial region of the intact telencephalon (**A**) of the juvenile *Oncorhynchus masou* and 1 week after the traumatic injury (**B**–**D**) of the telencephalon. (**A**) Localization of DC in intact pallium, immunopositive cells (inset, red arrows), radial glia (black arrows) and granules (white arrows) in the parenchymal zone (PZ); (**B**) DC+ cells (inset) and clusters (white dashed oval) of intensely DC-labeled cells (red arrow) and moderately DC-labeled cell (yellow arrow) in PVZ of Dd, 1 week after damage to the telencephalon, Vim+ granules (blue arrow); (**C**) post-traumatic expression of DC immunopositivity in Dl, DC+ heterogeneous complexes in PVZ and SVZ (an enlarged fragment is shown in the inset), alternating with moderately-labeled areas, accumulation of intensely DC-labeled cells (in a yellow dashed oval) other designations as in Figure 5B; (**D**) patterns of DC-immunopositivity after trauma in PVZ and SVZ Dm (inset), intensely labeled cells (red arrows), moderately labeled cells (yellow arrows), clusters of intensely labeled cells (in a yellow oval); (**E**) the quantitative ratio of DC+ cells in intact animals (control group) and 1 week after traumatic injury to the telencephalon (*n* = 5 in each group, * *p* ≤ 0.05; ** *p* ≤ 0.01; significant difference from control groups). Student–Newman–Cales test. Immunohistochemical labeling of doublecortin in combination with methyl green staining. Scale bar: in (**A**) 200 μm, (**B**–**D**) 100 μm.

In intact juvenile *O. masou*, a slight expression of doublecortin (DC) in cells was observed in the pallial region of the telencephalon (Figure 5A, Table 2). We identified single intensely labeled DC+ cells located in the PVZ (Table 2). Another type of immunolabeled structures was represented by radial fibers that extended from PVZ into the brain parenchyma (Figure 5A, inset). Unlike cells, radial fibers were poorly labeled. In PVZ, we found weakly or moderately DC+ cells located among DC cells (Figure 5A, Table 2).

Cells were labeled non-uniformly; in intensely labeled cells, the nucleus was not visualized against the background of stained cytoplasm (Figure 5A, inset). With less dense DC labeling, an immunonegative nucleus was clearly visible, and IHC labeling products were usually concentrated in the peripheral layers of the cell cytoplasm (Figure 5A). Intensely labeled granules with subcellular localization were detected during the IHC labeling of DC in PVZ and SVZ cells, as well as in deeper layers of PZ (Figure 5A).

The morphological analysis of cells labeled with DC showed that most cells in the pallial region of the telencephalon are heterogeneous; their parameters are shown in Table 2. DC immunolabeling was detected in PVZ of the telencephalon and was not found in the deep parenchymal regions of the *O. masou* pallium (Figure 5A). The results of densitometric studies showed significant differences in the values of optical density in intensively, moderately, and weakly DC+ cells (Table 2).

Thus, DC expression in the juvenile *O. masou* pallium was detected in cells and fibers of areas with constitutive neurogenesis, and was not observed in the areas containing differentiated cells. Nevertheless, in the areas containing mature neurons, single intensely DC-labeled granules were identified, which in most cases had intracellular localization.

At 1-week post-injury, significant changes were observed in the brain of juvenile *O. masou*. In all areas of the pallial zone of the telencephalon, a significant increase in the number of DC+ cells and the intensity of their labeling was recorded. After the damage to DC+, the cells located in intact fish only in PVZ were observed in PVZ, SVZ, and superficial parenchymal layers (Figure 5B–D). Three types of cells were identified in Dd, Dl, and Dm: intensely DC-labeled, moderately DC-labeled, and DC-negative cells. In intensely labeled DC+ cells, the cytoplasm was uniformly stained, and the nucleus was not visualized or poorly detected. In moderately labeled DC+ cells, IHC reaction products were concentrated at one or both poles of the cell, and the immunonegative nucleus in such cells was clearly visualized.

In Dd DC+, the cells lined the proliferative zone in a dense layer (Figure 5B). Intensely labeled DC+ polygonal and elongated cells were identified in PVZ (Table 2). Intensely labeled DC+ oval cells were identified in PVZ, both single and forming continuous layers up to 250 μm in length (Figure 5B, inset, Table 2).

Areas containing intensely DC+ labeled cells alternated, with regions comprising moderately DC+ labeled cells with large eccentrically located immuno-negative nuclei (Figure 5B, Table 2). In such cells, DC expression was less intense than in PVZ cells. Another type was represented by the smallest single, intensely DC+ labeled, and round-shaped cells. A dense homogeneous layer of DC cells corresponding to neuroepithelium was located above the layer of DC+ cells (Figure 5B). Such cells were organized into a single-row formation; some cells contained apically located cilia (Figure 5B). Single moderately immunopositive cells were localized in PZ (Figure 5B). 

Outside the PVZ, DC+ cells were located at a considerable distance from each other and did not form clusters; such cells had an oval shape and were medium in size (Table 2). The cells with DC labeled cytoplasm were founded; in such cells, the immunonegative nuclei were well visualized (Figure 5B, Table 2). In the deep parenchymal layers of Dd, we found single small intensely labeled cells (Table 2), morphologically similar to PVZ elements. In some cases, ectopic DC+ elements crescent shapes were visualized (Figure 5B). In most cases, labeled granules were located at one of the poles of the cell; in some cases, multiple granules were noted (Figure 5B). In intact animals, the average number of DC+ cells was 7 ± 1; at 7 days post-injury, the number of cells in Dd increased to 112 ± 11 cells. Thus, as a result of the mechanical injury, the number of DC+ cells in Dd increased 16-fold (Figure 5E).

In Dl, juvenile *O. masou* also had a layer of intensely labeled DC cells in PVZ (Figure 5C). DC+ cells formed in Dl segments up to 400 μm in length. Intensely labeled cells were represented by two size groups of cells (Table 2). In SVZ, single moderately labeled cells of three types were distinguished (Table 2). Small intensely labeled DC cells and many intensely immunolabeled granules of subcellular and extracellular localization were identified in PZ (Figure 5C, Table 2).

DC+ cells in Dm, unlike Dd and Dl, did not form a layer in the periventricular region (Figure 5D). Dm was dominated by moderately labeled cells of two-dimensional types (Table 2), whose cytoplasm was labeled at the poles, and nuclei were well visualized. In PVZ, we identified single intensely labeled cells of two types (Table 2), located at the outer border of the telencephalon and *pia mater* (Figure 5D). Another cell type was represented by elongated, moderately DC-labeled cells, with a large centrally located negative nucleus (Table 2). In some of these cells, a long radially oriented DC process was clearly visualized (Figure 5D). Small, intensely labeled DC cells were also identified in SVZ; single or paired labeled granules were found occasionally (Figure 5D, Table 2). DC+ intracellular inclusions often had an irregular shape, in which not only centrally located granules were visualized, but also processes of irregular shape (Figure 5D).

Another DC immunolocalization pattern was detected in the intermediate Dd/DM zone, between Dd and Dm (Figure 5D). Here were found elongated tangentially migrating cells with intensely labeled cytoplasmic inclusions (Figure 5D). Larger cells contained multiple labeled granules at one of the poles and single ones at the opposite. In the intermediate Dd/DM zone, there were dense clusters of tangentially migrating intensively labeled cells, with a length of 220 μm (Figure 5D).

The topological organization of DC+ and DC- neurons was observed in Dm. We revealed radial direction of cells migration, elongated morphology of most cells, and the lack of signs of cells differentiation. The above facts allow us to attribute DC+ cells to a migratory population of neuroblasts. Thus, after the mechanical injury, the number of cells in Dm increased 20-fold (Figure 5E). The increase in the number of DC+ neuroblasts was both due to the growth in number of small single intensely labeled cells, and due to the increase in moderately labeled cells containing DC+ granules.

## 4. Discussion

### 4.1. Expression of Brdu in the Juvenile Masu Salmon Pallium under Normal Conditions and after Traumatic Injury

A study of adult neurogenesis in fish began relatively recently [24,25]. BrdU is a modified nucleoside that is easily integrated into the structure of proliferating cells. Currently, there is little data of experiments with the incorporation of BrdU on several fish species [19,26]. It has been shown that in the *Austrolebias* telencephalon, proliferating cells are replaced by newborn neurons within 24 h after BrdU administration [27,28], which is probably due to the short life cycle of this fish. For other fish species, longer periods of neurogenesis were reported: 3 days in pallium and 15 days in the olfactory bulbs of *D. rerio* [24]; 7 days in the telencephalon of *Nothobranchius furzeri* [29]. A high but changing number of cells in the adult brain was found in tilapia *Oreochromis mossambicus*, at 100 days after BrdU administration [30].

As a result of the study, a heterogeneous population of BrdU+ cells and nuclei was revealed in the pallium of juvenile *O. masou*. The study has also shown BrdU-immunolocalization in the Dd, Dl, and Dm pallial zones of the telencephalon of juvenile *O. masou*. Among BrdU-immunolabeled elements, intensely labeled cells and nuclei were revealed according to the Traniello classification [31]. The size of the nuclei, according to the classification of Candal [32], was up to 3–3.5 μm. In the pallium of intact juvenile *O. masou*, intensively similar BrdU+ nuclei of a similar size were revealed in all zones (Table 1). The largest numbers of small intensely BrdU-labeled cells were detected in Dd and the smallest were in Dm, which suggests the largest contribution to the constitutive neurogenesis of the dorsal and the smallest contribution to the medial pallial regions. The results of BrdU-immunolabeling are consistent with previously obtained PCNA immunolabeling data for the pallium of young *O. masou* [15]. According to data published in literature, PCNA labels the additional DNA polymerase (delta), retained in the cell within 24 h after the completion of mitosis [33], but the level of PCNA activity decreases by 30% [34]. BrdU immunolabeling allows the diagnosis of cells and nuclei that are in the S-phase of the cell cycle, while PCNA labeling can be used to visualize a larger population of cells, that are both in proliferation and recently emerged from the cell cycle [34].

BrdU-labeling in Dl and Dm of the intact pallium of juvenile *O. masou* showed heterogeneous populations of BrdU+ cells, in contrast to Dd. In Dl, patterns of tangential surface migration of elongated BrdU+ cells were detected; in Dm, the radial migration of BrdU+ cells from PVZ to SVZ was revealed. After PCNA labeling in young *O. masou*, it was found that more PCNA+ cells are concentrated in Dm than in Dd and Dl [15]. However, the results of the present study show that among the proliferating population of pallium cells, in the S-phase are mainly cells in Dd. Cells at other stages of the mitotic cycle, including a state of migration, are located in Dm. Both during PCNA labeling and the experimental administration of BrdU into Dm of juvenile *O. masou*, immunopositive cells and nuclei were detected in SVZ and PZ. However, while BrdU labeling revealed a few isolated cells in the PZ and SVZ, whereas after PCNA immunolabeling, the patterns of cells with proliferative activity proved to be more numerous [15].

Features of the distribution of BrdU+ cells in intact juvenile *O. masou* indicate a high intensity of the constitutive neurogenesis processes, that occur not only in the matrix proliferative zones of pallium located in PVZ, but also in the deeper SVZ and PZ of the telencephalon. The presence of BrdU+ cells in SVZ and PZ of *O. masou* confirms the data obtained on the *Apertonotus leptorhynchus* [35], according to which, single proliferating cells were also found in this fish in the telencephalon parenchyma. The data obtained on juvenile *O. masou* can be interpreted by taking into account the features of the ontogenetic development of juvenile salmon fish by fetalization, which is characterized by a slowdown in the ontogenesis rates of certain organs or their systems, as a result of which the adult organism retains the embryonic state of the respective characters [15]. The fetalization processes are superimposed on the stage of active growth, in which the morphogenesis processes are most clearly and fully expressed. The group of salmon fish is a phylogenetically ancient branch of vertebrates, characterized by a high concentration of undifferentiated elements, not only in the matrix zones, but also in the brain parenchyma. Our study of BrdU+ cells in PVZ, SVZ and PZ of the pallium of the intact juvenile *O. masou* confirms this opinion and provides evidence of the high proliferative potential of the pallial cells in the telencephalon of the growing juvenile *O. masou*.

The traumatic injury caused the labeling patterns of BrdU+ cells to change, both in the composition of matrix PVZ and in the deeper SVZ and PZ of the pallium of juvenile *O. masou*. Small clusters of BrdU+ cells appeared in the PVZ of Dd and Dl, as well as single BrdU+ cells in deeper SVZ and the PZ of Dm. Moreover, we observed an increase in the number of labeled cells in PVZ and the appearance of additional types of BrdU+ nuclei that were not detected in intact animals. The appearance of clusters of BrdU+ cells indicates the synchronization of proliferative activity in cells, which are probably descendants of the glial type of aNSCs. The results are consistent with data on zebrafish, in which a glial type of aNSCs was detected in the dorsal telencephalon [5]. The source of such cells is embryonic radial glial cells [6], which do not directly produce neurons, but first produce intermediate progenitor cells [7]. Zebrafish studies have shown that after trauma the glial progenitor cells begin to divide synchronously, expanding the pool of newly formed progenitor cells involved in the repair process, some of which have the ability to migrate over significant distances [36]. Nevertheless, the studies of reparative neurogenesis patterns in other adult animals and the involvement of glial precursors in adult neurogenesis need further development, since they do not reflect the complexity of vertebrate aNSCs.

Based on the fact that a part of the heterogeneous population of presumptive ventricular NPCs of *O. masou* juveniles express radial glia markers, while the others do not, it is relevant to assume that new neurons in the pallium of juvenile *O. masou* can arise from a neuronal and non-neuronal population of progenitor cells. This assumption is consistent with the data obtained on adult *D. rerio* [37], in whose central nervous system the expression of embryonic genes is preserved: GFAP, S100b, BLBP, GLAST, and vimentin [38]. RG is present in the telencephalon of juvenile *O. masou*, lining the everted ventricular surface of the pallium and forming new neurons during various periods of postembryonic ontogenesis [39], which agrees with the earlier monitoring observations of the postembryo nic development of *D. rerio* [10].

The pattern of GFAP immunolabeling in the pallium of juvenile *O. masou* after injury was significantly different from the GFAP labeling in intact animals. Instead of single GFAP+ cells, heterogeneous cell clusters, additional radial glia fibers, and single small intensely GFAP-labeled cells in the parenchyma appeared in Dd and Dl. All of these GFAP+ elements appeared de novo, as a result of the activation of the resident aNSCs glial type and their subsequent slow proliferation in response to damage. This was most pronounced in juvenile *O. masou* in Dl, where we discovered heterogeneous clusters, including both GFAP-positive and GFAP-negative cells and radial glia fibers, as well as GFAP-positive granules. We believe that these GFAP-immunopositive structures are reactive neurogenic niches containing a glial type aNSCs that occur in response to injury. Studies on zebrafish pallium have revealed radial glial aNSCs and intermediate precursors [3]. The source of aNSCs is embryonic radial glia [6] produced by intermediate progenitor cells [7], which are highly heterogeneous in terms of active cyclicity or rest [8] and expressed molecular markers [9,10].

Studies on juvenile *O. masou* showed that BrdU+ clusters of cells and nuclei appear as a result of injury, not only in the primary proliferative zone (PVZ), but also in the deep SVZ and PZ of the telencephalon, which indicates the high reparative potential of the pallium of juvenile *O. masou*. After the injury, additional proliferative activity was induced in cells and nuclei that did not show BrdU-labeling in control animals (Table 1). The different intensities of BrdU labeling in intact animals and its increase up to 15% in different areas of the pallium after the injury indicates that proliferating cells may not be time-synchronized. The morphological heterogeneity of BrdU+ cells and nuclei in various areas of pallium post-injury may indicate various sources of proliferating cell origin. Studies on *Danio rerio* have shown that a damage results in the reactivation of specific genetic programs in resident aNSCs, leading to the activation of proliferative processes [36]. We believe that after an injury to the telencephalon *O. masou*, aNSC activation processes also occur, which result in a local increase in proliferative activity in cells of different types and possibly of different origin. BrdU labeling in larger cells not detected in PVZ of the intact *O. masou* may indicate the reprogramming and transdifferentiation of such cells as a result of injury. This hypothesis is supported by the results of recent studies on zebrafish, according to which mature neurons, when damaged, can transdifferentiate and form a pool of proliferating cells [40]. No similar direct transformation and/or transdifferentiation was previously recorded from the adult brain of other fish species, which indicates either its species-specific nature or direct transformation that has not yet been found in the brain of other species, due to the lack of a suitable methodology, in particular, intravital imaging.

### 4.2. Expression of Gfap and Vimentin in the Brain of Fish under Normal Conditions and after Traumatic Injury

The use of molecular markers to verify NSCs, neuronal precursors, and differentiated forms of neurons in the brain of adult mammals and other vertebrates has some contradictions. Thus, GFAP and vimentin are considered as markers of astrocytic glia in the vertebrate brain [22]. However, no typical astrocytes have been found in the fish brain, while a limited population of cells without processes or with weakly expressed apical processes [41], as well as widespread radial glia cells [42], is identified. According to some data [43], GFAP and nestin label a limited population of NSCs in the mammalian brain. Thus, it is currently not clear whether neural stem cell markers are common for mammals and other vertebrates.

Studies on the grey mullet *Chelon labrosus* have shown that the level of expression of vimentin and GFAP varies throughout life. In particular, it was found that in this species the expression of GFAP increases with age, but the expression of vimentin, in contrast, decreases [22]. GFAP immunolabeling in the early stages of development of *C. labrosus* revealed cell bodies, as well as processes of astrocytes and tanycytes, while the expression in radial glia cells was determined at later stages of development [22]. In studies of the pallial zone in the telencephalon of juvenile *O. masou*, no cells with a tanycytes-like morphology and/or typical process astrocytes were detected in the proliferative zones. However, single intensely labeled GFAP cells with no signs of differentiation were identified. In this regard, we believe that the pallium regions containing Vim and GFAP immunopositivity correspond to zones with high neuronal plasticity, containing adult tissue-specific neuronal precursors that determine high neurogenic potential in the postembryonic development period of juvenile *O. masou*. This is consistent with data from studies of the mammal CNS, which have shown that a certain population of astroglial cells, in particular, radial glia of ependyma and subependymal regions, can produce neurons, as well as astrocytes and oligodendrocytes, thus, indicating the specialization of such cells as aNSC [2,5]. In mammals, RG is retained in the forebrain [44]. However, in neurogenic niches activated by damage, RG is absent. In fish brain, reactive astrogliosis does not develop, since most areas surrounding the injury zone contain RG cells instead of astrocytes [3,40]. Moreover, in the adult *D. rerio* brain, RG, like astroglia in mammalian brain, shows characteristic signs of reactive gliosis immediately after injury [45,46]. 

Based on the fact that part of the heterogeneous population of presumptive ventricular NPCs of juvenile *O. masou* express RG markers, while the other does not, it is relevant to assume that new neurons in the pallium of *O. masou* can arise from a neuronal and non-neuronal population of progenitor cells. This assumption is consistent with the data obtained on the adult *D. rerio* [37], in the central nervous system of which the expression of embryonic genes is retained: GFAP, S100b, BLBP, GLAST, vimentin [38]. RG is present in the telencephalon of juvenile *O. masou*, lining the everted ventricular surface of the pallium and forming new neurons during various periods of ontogenesis [39]. These data correspond to earlier monitoring observations of the postembryonic development of *D. rerio* [10].

The pattern of GFAP immunolabeling in the pallium of juvenile *O. masou* after injury was significantly different from the GFAP labeling in intact animals. Instead of single GFAP+ cells, heterogeneous cell clusters, additional radial glia fibers, and single small intensely GFAP-labeled cells in the parenchyma appear in Dd and Dl. All of these GFAP+ elements appear de novo as a result of activation of the resident aNSCs (glial type), and their subsequent slow proliferation in response to damage. It was presented in Dl of juvenile *O. masou*, where we discovered heterogeneous clusters, including both GFAP-positive and GFAP-negative cells and radial glia fibers, as well as GFAP-positive granules. We believe that these GFAP-immunopositive structures are reactive neurogenic niches containing a glial type of aNSCs that occur in response to injury. Studies on zebrafish pallium have revealed radial glial aNSCs and intermediate precursors [3]. The source of aNSCs is embryonic RG cells [6], which are produced by intermediate progenitor cells [7]. The population of intermediate progenitors are highly heterogeneous in terms of active cyclicity or rest [8] and express corresponding molecular markers [9,10].

Vimentin is an intermediate filament protein, which is most commonly found in astroglial cells and immature astrocytes [47,48]. Immunohistochemical studies of ependymal cells and radial glia, present in large numbers in the brain of bony fish, showed the presence of GFAP [49] and vimentin [50] in them. Vimentin sequencing of bony fish and amino acid sequence analysis revealed a high degree of its homology with human protein [50,51]. The ratio of neuron and gliospecific proteins, labeling populations of proliferating progenitor cells in the matrix zones of the telencephalon and cerebellum in different fish species, is not the same [3,52]. Vim-positive cells were detected in the telencephalon of different bony fishes. In the zebrafish pallium, glial phenotype cells labeled by vimentin are detected [50]. However, in the intact pallium of juvenile *O. masou*, Vim-positive cells corresponded to the neuroepithelial phenotype. In early grey mullet *C. labrosus* larvae, weak immunopositivity for vimentin was revealed in the RG cells, in the ventral region of the telencephalon. In later *C. labrosus* larvae, Vim expression is more pronounced, and in large larvae, it reaches the highest level of activity [22]. In the pallium of juvenile *O. masou*, vimentin expression is low, which is consistent with data on the decrease in vimentin expression in the telencephalon of *C. labrosus*. However, in adult mullet, weakly Vim-immunopositive cells are localized mainly in the subpallial, rather in the pallial, zone of the telencephalon around blood vessels [22].

After telencephalic injury of juvenile *O. masou* in Dd, an increase in vimentin expression cells was revealed in PVZ, which is consistent with data for zebrafish [24,53]. Similar to GFAP labeling, clusters of highly Vim-positive cells and numerous less intensely Vim-labeled Dd cells, as well as single labeled cells in the brain parenchyma, were revealed in young *O. masou* after traumatic injury. Extensive regions containing intensely Vim-labeled cells and radial glia, as well as pairs of labeled cells in different layers of the parenchyma, were found in Dl. As in the case of GFAP, the labeling of Vim in Dm of juvenile *O. masou* was characterized by the least pronounced structural and quantitative changes in the number of immunopositive cells.

After the traumatic injury, vimentin labeling was characterized by a significant (*p* < 0.05) increase in the number of immunopositive elements: cells and granules in comparison with the intact expression of Vim in pallium *O. masou*. An increase in the number of Vim+ cells was characteristic of PVZ of Dd and Dl pallium, clusters of Vim+ cells without processes appeared in Dd (Figure 4B), and patterns of Vim+ RG clusters appeared in Dl (Figure 4C). We believe that as a result of the traumatic process in the pallium of *O. masou*, an additional pool of Vim+ aNSCs with glial phenotype and their descendants of the NPCs were activated, aiming to eliminate the effects of the injury. The number of Vim+ granules in PVZ and SVZ also increased; their distribution density increased significantly compared to the control. Enhanced vimentin expression at the intracellular level was accompanied by the labeling of Vim+ granules in dense reactive NE cell conglomerates, neuroepithelial type in Dd and Dm, and radial-glial in Dl (Figure 4B–D). Significant rearrangements of the neuroepithelial layer were observed in PVZ, expressed as local hypertrophy of areas with neuroepithelial cells (Figure 4B), inside which the intracellular presence of Vim+ granules was also detected. The results obtained from the pallium of juvenile *O. masou* correspond to the results of damage to the zebrafish pallium [45,53], which showed that traumatic damage is accompanied by the development of reactive gliosis, during which astrocytes show increased expression of intermediate filament proteins, such as GFAP and vimentin, and also alter the expression of certain genes [45,53]. Damage to the zebrafish pallium enhances the expression of GFAP, vimentin, nestin, and calcium-binding protein S100b in RG, with hypertrophied processes [45]. Similar structural changes in RG were revealed by us, after GFAP labeling of the tegmentum of juvenile chum post-injury [14].

The revealed structural changes in the expression of GFAP and vimentin in the pallium of juvenile *O. masou* resemble the appearance of gliosis in mammals except, however, scar formation that is not expressed in the pallium of *O. masou*, which agrees with data on zebrafish [45,53]. According to the data of Takeda [54], GFAP+ processes of fish RG repair damaged axons to a greater extent than they form a scar. A detailed study of the cellular composition and patterns of extracellular immunolocalization of vimentin in the pallium of juvenile *O. masou* after damage indicates a multiple increase in the processes of constructive metabolism. Another important consequence is the multiple increases in Vim+ NCPs generated after injury. The presence of local clusters of intensely labeled cells of similar size and shape indicates the proliferation of progenitor cells, increasing the total number of cells involved in the reparative process. Thus, post-injury vimentin expression patterns correspond to GFAP expression and indicate a similar increase in the expression of intermediate filament proteins in the damaged pallium of juvenile *O. masou*. After injury, Vim+ cells with neuroepithelial morphology were identified in Dd and Dm, and radial glia-like morphology was in Dl.

### 4.3. Expression of Doublecortin in the Intact Brain of Fish and after Traumatic Injury

Doublecortin is a microtubulin-associated protein, necessary for the migration of young neurons in the vertebrate brain [55] and is used as a marker of a migratory neuroblasts population. Immunohistochemical studies conducted on more than 70 mammalian species showed the presence of DC as a marker of hippocampal neurogenesis in adult animals [56]. Adult neurogenesis was also investigated in the brain of birds, *Gallus gallus*, *Columbia livia* and *Streptopelia risoria*, in which PCNA and DC immunopositive cells were found in the lumen of the cerebral ventricle, labeling newly formed neurons in the brain of adult animals [57]. 

Doublecortin is a protein expressed by immature neurons associated with microtubules [23]. The migration of neurons is a critical process in the development of the nervous system. A unique expression pattern of doublecortin allows its use as a marker of neurogenesis in adult animals, and mutations in this protein disrupt neuron migration, leading to the development of pathological changes [58]. Although DC is able to modulate and stabilize microtubules to ensure efficient migration of neuroblasts, the mechanisms involved in performing these functions remain poorly understood. The unstable interaction between DC and microtubules destabilizes the cytoskeletal organization, leading to impaired cell migration [23]. The expression of doublecortin (but not the expression of mitotic markers) persists for some time in young terminally differentiated neurons [59]. 

Doublecortin is expressed by new-generated and migrating neurons in the intact pallium of juvenile *O. masou*, localized both in the cytoplasm and in the nuclei of cells; its presence provides many intracellular processes. The expression of DC may be important in axon growth and/or synaptogenesis in the adult neurons [60], as well as in the dendrite growth cone [55]. The expression of doublecortin is retained in postmitotic neurons and coincides with the expression of calretinin [61].

In our studies, in the pallial region of the telencephalon of intact juvenile *O. masou*, intense individual DC+ cells without radial processes, as well as DC+ granules, were detected. A study of DC localization in the pallium of *O. masou* showed low protein expression in the cells of primary neurogenic zones, with a predominance of the radial phenotype of the precursors in Dd. Compared to Vim- and GFAP-immunolocalization, the number of DC+ cells is reduced in pallium PVZ, which probably indicates a relatively low amount of postmitotic neuroblasts in PVZ. However, in different areas of intact pallium, different contents of DC+ granules were detected. We suggest that DC expression in the form of granules in the pallium of *O. masou* may be necessary to ensure the neuronal plasticity of adult neurons.

Doublecortin is necessary for neurogenesis in adult mammals [60] and is a marker of newly generated neurons [61]. In *Nothobranchius furzeri*, DC labels newly generated neurons in the pallium, indicating similar functions of DC in mammals and fish. A mammalian study showed that neurons generated in adulthood are functionally active and integrate into existing neuronal networks, and are suggested to be involved in some aspects of behavioral plasticity [62]. In bird studies, DC-expressing cells have unipolar, bipolar, or multipolar morphology with long processes [57]. A particularly high concentration of DC+ cells was characteristic of the pallial regions of the telencephalon, with the caudal regions of the pallium being more intensely labeled as compared with the rostral ones [57,59]. 

The results of our studies on juvenile *O. masou* are generally consistent with data obtained on birds and other vertebrates. Thus, we associate the presence of DC-positive cells in the pallial region with the processes of constitutive postembryonic neurogenesis and neuronal plasticity, which is consistent with the results of studies on the pallium of *D. rerio* [25] and *N. furzeri* [29]. The results of studies on the pallium of juvenile *O. masou* also confirm the simultaneous process of radial migration of newborn cells from the proliferation zone to the surrounding subpallial cell masses, which is consistent with previously obtained data on other fish species [25,29]. 

After injury, DC immunolabeling in the pallium of juvenile *O. masou* underwent significant changes. Currently, there are few papers related to the use of DC as a marker of reparative neurogenesis in the mammalian brain during CNS injury [23]. In the pallial part of the telencephalon of *O. masou*, in comparison with the control, we observed intense DC-labeling of cells in the subventricular parts of the brain. A characteristic feature of DC-labeling detected after an injury is a pronounced pattern of cell migration from the neurogenic zone of pallium into the parenchyma of the brain. This confirms the simultaneous process of radial migration of numerous neuroblasts, newly formed as a result of the traumatic process. An extensive population of the *O. masou* pallium cells containing doublecortin after injury included moderately DC-labeled neuroblasts containing cytoplasmic DC+ inclusions, as well as small, intensely DC-labeled elements. The appearance of intensely DC-labeled cells in the brain parenchyma, in our opinion, may indicate the activation of resident NSCs pools activated as a result of the traumatic process, which is consistent with data on mammals [23].

After the injury, the occurrence of patterns of completed mitoses, which are a pair of closely spaced, intensely DC-labeled cells located in the deep layers of the parenchyma outside the proliferative zones, is characteristically observed in the parenchymal zone of the pallium (Figure 5B). Similar structures are absent from the pallium of intact juvenile *O. masou*. Small DC+ cells of parenchymal localization may correspond to the aNSCs population activated as a result of the traumatic process. This is consistent with the data obtained on mammalian mesenchymal stem cells, according to which very small cells, having a size no larger than 5 μm, exhibit a high proliferative potential and the highest proliferative activity [63]. We believe that the occurrence of such cellular forms in the brain of juvenile *O. masou* is one of the consequences of injury. In Dm, in contrast to Dd and Dl, we observed highly immunopositive cells, both in the pallial parenchyma and in the territory of the PVZ. In Dm, the most numerous DC-labeled migratory cells with moderate DC immunoreactivity were identified. Thus, we believe that, an additional pool of highly active aNSCs, as well as patterns of mass migration of newly formed DC+ neuroblasts migrating from neurogenic zones to the deep layers of the parenchyma, are formed in the *O. masou* pallium as a result of the traumatic process.

## 5. Conclusions

The distribution of BrdU+ cells in intact juvenile *O. masou* indicates a high intensity of the processes of constitutive neurogenesis in the pallium. The maximum number of BrdU+ cells and nuclei was detected in Dd; the minimum, in Dm (*p* < 0.05). After the traumatic injury of the telencephalon, an increase in the number of BrdU+ cells and nuclei and the intensity of their labeling was observed in all areas of the pallium of juvenile *O. masou*; significant differences from the control group (*p* < 0.05) were detected in Dm.

The studied molecular markers: vimentin, GFAP, and doublecortin in the pallium of intact juvenile *O. masou* allowed only single immunopositive cells to be identified. A subsequent analysis of these cells detected in the territory of the pallium matrix zones showed that they are mainly of the same type, small rounded and oval cells, detected in all variants of immunolabeling, which allows us to consider them as a persistent pool of neuronal and/or glial precursors present in the palial of the matrix zone of juvenile *O. masou*. BrdU-labeling data also indicate the presence of similar cells in Dl and Dm of intact animals, suggesting the presence of tissue-specific neuronal precursors of the glial phenotype in juvenile *O. masou*.

The GFAP immunolabeling pattern in the telencephalon of juvenile *O. masou* after injury significantly differed from the GFAP labeling in intact animals. Heterogeneous cell clusters, additional radial glia fibers, and single small intensely GFAP-labeled cells in the parenchyma appear in Dd and Dl. All of these GFAP+ elements appear de novo as a result of activation of the resident glial type aNSCs and their subsequent slow proliferation in response to a damage. This is most pronounced in Dl, where heterogeneous clusters were detected, including both GFAP-positive and GFAP-negative cells, radial glia fibers, as well as GFAP-positive granules. We believe that these GFAP-immunopositive structures are reactive neurogenic niches containing glial aNSCs that occur in response to damage. A detailed study of the cellular composition and patterns of extracellular immunolocalization of vimentin in the pallium of juvenile *O. masou* after damage indicates a multiple increase in the processes of constructive metabolism. Another important consequence is the multifold increase in the pool of Vim+ NPCs generated after trauma. The presence of local clusters of intensely BrdU-labeled cells of similar sizes and shapes indicates the proliferation of progenitor cells, increasing the total number of cells involved in the reparative process. Thus, post-traumatic vimentin and GFAP expression patterns indicate a similar increase in the expression of intermediate filament proteins in the damaged pallium of juvenile *O. masou*. After injury, Vim+ cells with neuroepithelial morphology were identified in Dd and Dm; the glial glia-like morphology, in Dl.

In the pallium of *O. masou* after injury, intense DC-labeling of cells was observed in the periventricular layers of the brain. A characteristic feature of DC labeling after injury is the appearance of the pattern of cell migration from the neurogenic zone of the pallium deep into the brain parenchyma. This confirms the simultaneous process of radial migration of numerous new generated neuroblasts as a result of the traumatic process. The appearance of intensely DC-labeled cells in the brain parenchyma indicates the activation of resident NSCs after the traumatic process. In the parenchymal zone after trauma, the occurrence of closely spaced pairs of intensely DC-labeled cells located outside the proliferative zones is characteristic. Thus, as a result of the traumatic process, an additional pool of highly active aNSCs in the pallium, as well as patterns of mass migration of newly formed DC+ neuroblasts migrating from neurogenic zones to the deep layers of the parenchyma, are formed in *O. masou*.

The obtained data actualize various questions for further studies of adult neurogenesis in the brain of Pacific salmon; in particular, how does the ratio of neuroepithelial and glial precursors change during the adulthood of *O. masou*? How does the rate of proliferation of pallium cells change in the later stages of postembryonic development of the *O. masou* brain, because the brain size of an adult *O. masou* is many times larger than that of a juvenile? From what types of neural and/or glial precursors during the *O. masou* life circle is the structure of various subregions of the telencephalon formed? Answers to these questions can significantly advance current knowledge about the neurogenesis of salmon fish, as convenient models for brain development research.

## Figures and Tables

**Table 1:** Morphometric parameters of BrdU+ cells and nuclei (M ± SD) in the pallial zone of the telencephalon of intact juvenile masou salmon (*Oncorhynchus masou*) and after injury.

	Intact Animals		Damaged Telencephalon		
Brain Areas Cell Type	Cell/Nuclei Size, µm	Optical Density, UOD	Cell Type	Cell/Nuclei Size, µm	Optical Density, UOD
Dd Undifferentiated cells	4.5 ± 0.5/2.8 ± 1.0	151.7 ± 6.2	undifferent. oval	4.3 ± 0.3/2.8 ± 0.4 5.6 ± 0.2/2.8 ± 1.0	177.1 ± 5.4 177.6 ± 3.6
nuclei	3.7 ± 0.2/2.3 ± 0.1	145.4 ± 2.3	nuclei	3.6 ± 0.3/2.5 ± 0.5 2.8 ± 0.2/2.0 ± 0.3	169.2 ± 5.1 168.4 ± 3.5
Dl Undifferentiated cells	4.6 ± 0.3/3.4 ± 0.5	147.8 ± 1.9	undifferent.	4.4 ± 0.3/2.7 ± 0.4	174.4 ± 5.1
Oval	5.6 ± 0.4/3.9 ± 0.4 7.6 ± 0.5/5.3 ± 0.9	151.9 ± 4.5 144.2 ± 3.3	oval	5.9 ± 0.5/4.3 ± 0.8	180.5 ± 3.0
nuclei	3.4 ± 0.4/2.7 ± 0.2	145.8 ± 4.6	nuclei	3.5 ± 0.3/2.4 ± 0.2 2.4 ± 0.3/1.9 ± 0.4	169.5 ± 1.2 165.7 ± 3.1
Dm undifferentiated	4.2 ± 0.3/2.8 ± 0.2	135.4 ± 2	undifferent.	4.4 ± 0.2/3.1 ± 0.1	152.6 ± 1.8
oval	5.2 ± 0.2/3.1 ± 0.6 7.2 ± 0.6/3.4 ± 0.5	152.2 ± 3.6 153.7 ± 1.1	oval	-	-
nuclei	3.4 ± 0.3/2.5 ± 0.6	144.9 ± 8.6	nuclei	3.4 ± 0.2/2.5 ± 0.3 2.7 ± 0.1/2.3 ± 0.3	155.3 ± 3.7 151.7 ± 4.8

**Table 2:** Morphometric parameters of GFAP+, vimentin+ and doublecortin+ cells (M ± SD) in the pallial dorsal (Dd), lateral (Dl) and medial (Dm) zones of the telencephalon of intact juvenile masou salmon (*Oncorhynchus masou*) and after injury.

Intact Animals		Damaged Telencephalon					
Cell Size, µm/Localization	Optical Density, UOD	DD		DL		DM	
		Cell Size, µm/Localization	Optical Density, UOD	Cell Size, µm/Localization	Optical Density, UOD	Cell Size, µm/Localization	Optical Density, UOD
**GFAP**							
10.3 ± 1.3/6.6 ± 1.0 (PVZ) 12.3 ± 0.3/7.9 ± 0.9 (PVZ) 13.4 ± 0.3/9.7 ± 1.0 (PVZ)	+++ +++ +++	14.2 ± 1.1/9.3 ± 0.2 (PVZ) 16.4 ± 0.8/8.5 ± 1.3 (PVZ)	+++ +++	5.1 ± 0.8/4.5 ± 1.0 (PZ) 9.7 ± 0.2/6.5 ± 0.2 * (PVZ) 11.4 ± 0.3/6.1 ± 1.2 (PVZ) 12.9 ± 0.6/7.1 ± 0.8 (PVZ)	+++ +++ +++ +++	9.7 ± 0.2/6.6 ± 0.6 (PVZ) 11.7 ± 1.0/7.1 ± 1.7 (PVZ) 14.8 ± 0.3/9.2 ± 1.1 (PVZ)	+++ +++ +++
10.8 ± 1.3/6.9 ± 0.2 (PVZ) 12.8 ± 0.2/8.5 ± 1.3 (PVZ) 14.5 ± 0.2/10.1 ± 1.4 (PVZ)	++ ++ ++	11.9 ± 0.5/9.5 ± 0.3 (PVZ) 16.3 ± 0.2/9.7 ± 1.1 (SVZ) 18.8 ± 2.4/10.7 ± 2.1 (SVZ)	++ ++ ++	7.4 ± 0.4/5.8 ± 0.3 (PVZ) 8.1 ± 0.2/5.7 ± 0.5 * (SVZ) 9.8 ± 0.6/5.8 ± 0.5 * (PVZ) 13.0 ± 2.4/6.2 ± 0.4 (PVZ)	++ ++ ++ ++	12.5 ± 0.4/8.5 ± 1.4 (PVZ) 13.5 ± 0.3/8.3 ± 1.9 (PVZ) 14.7 ± 0.7/9.6 ± 1.8 (PVZ)	++ ++ ++
**Vimentin**							
9.2 ± 0.5/8.6 ± 0.4 (PVZ) 10.8 ± 0.6/7.4 ± 1.3 (PVZ) 13.2 ± 0.7/7.5 ± 1.7 (PVZ)	+++ ++ +++	3.2 ± 0.6/2.1 ± 0.3 (PZ) 8.6 ± 1.3/5.9 ± 0.9 * (PVZ) 10.5 ± 0.7/6.8 ± 1.3 (PVZ)	+++ +++ +++	5.5 ± 1.6/3.9 ± 1 (PZ) 11.2 ± 0.4/8.0 ± 0.3 (PVZ) 12.3 ± 0.3/8.2 ± 0.6 (SVZ) 17.3 ± 0.3/8.4 ± 1.0 (PVZ)	+++ +++ +++ +++	6.5 ± 0.5/4.0 ± 0.8 (PVZ) 9.3 ± 0.3/5.2 ± 1.1 (PVZ)	++ ++
9.1 ± 0.4/7.8 ± 0.5 (PVZ) 10.6 ± 0.2/7.4 ± 0.7 (PVZ) 11.5 ± 0.5/7.7 ± 1.5 (PVZ)	++ + +	9.0 ± 0.7/6.5 ± 0.9 (PVZ) 11.7 ± 1.0/8.0 ± 0.5 (PVZ)	++ ++	9.0 ± 0.2/6.6 ± 1.7 * (SVZ) 11.2 ± 0.6/7.3 ± 0.3 (PVZ) 13.1 ± 0.2/8.8 ± 1.1 (PVZ)	++ ++ ++	5.9 ± 0.4/3.7 ± 0.4 (SVZ) 7.7 ± 0.5/6.0 ± 0.7 * (PVZ) 10.1 ± 0.8/6.5 ± 0.9 (SVZ)	+ + +
**Doublecortin**							
8.6 ± 1.4/5.0 ± 0.9 (PVZ) 10.8 ± 0.5/6.3 ± 0.4 (PVZ) 13.0 ± 0.6/6.9 ± 2.4 (PVZ)	+++ ++ ++	4.6 ± 0.7/3.5 ± 1.1 (PZ) 14.3 ± 0.2/9.9 ± 0.9 (PVZ) 16.8 ± 0.5/10.9 ± 1.5 (PVZ) 21.6 ± 1.4/11.7 ± 2.2 (PVZ)	+++ +++ +++ +++	5.4 ± 1.3/4.2 ± 1.0 (PZ) 13.9 ± 1.2/10.1 ± 0.9 (PVZ) 16.2 ± 0.8/11.4 ± 0.8 (PVZ)	+++ +++ +++	5.2 ± 0.8/3.7 ± 0.6 (PZ) 9.4 ± 0.6/6.6 ± 0.8 (SVZ) 11.6 ± 0.8/6.7 ± 0.9 (PVZ)	+++ +++ +++
9.1 ± 1.2/6.9 ± 1.0 * (SVZ) 10.9 ± 0.3/6.6 ± 0.8 (PVZ) 12.3 ± 0.4/7.6 ± 1.6 (PVZ) 16.1 ± 0.8/9.5 ± 2.4 (PVZ)	++ + + +	12.0 ± 0.7/9.8 ± 1.8 (PVZ) 13.9 ± 0.4/10.1 ± 2.2 (SVZ) 15.5 ± 0.3/11.7 ± 1.8 (SVZ) 18.4 ± 0.8/13.4 ± 1.7 (SVZ)	++ ++ ++ ++	14.3 ± 0.3/13.1 ± 2.0 (PVZ) 16.5 ± 0.5/11.8 ± 0.9 (SVZ) 19.0 ± 1.0/12.0 ± 0.6 (SVZ)	++ ++ ++	10.3 ± 0.8/8.7 ± 1.5 (SVZ) 12.1 ± 0.4/8.1 ± 1.2 (SVZ) 15.3 ± 0.9/8.1 ± 1.5 (PVZ)	++ ++ ++

Optical density (OD) in CBS+ cells was categorized by the following scale: high (180–130 UOD, corresponding to +++), moderate (130–80 UOD, corresponding to ++) weak (80–40 UOD, corresponding to +), and low (less than 40 UOD, corresponding to –); the initial OD value was measured on the control mounts. Large- and small-sizes of neurons are shown through a slash. Cells were morphologically classified according to a previously developed scheme (Pushchina et al. 2017). * indicates radial glia that appear in the intact juvenile masu salmon *O. masou* telencephalon and/or after injury.
